# Fetal Congenital Complete Heart Block: 2 Success Stories Through Multidisciplinary Care

**DOI:** 10.1016/j.jaccas.2025.105158

**Published:** 2025-09-24

**Authors:** Mohamed Aashiq Abdul Ghayum, Jenna Schermerhorn, Hayley Hancock, Lindsey Malloy-Walton, Steven Olsen, Laura Vricella, Kelsey Brattrud, Melanie Kathol, Rita France, Maria Kiaffas

**Affiliations:** a Ward Family Heart Center, Children's Mercy Hospital, Kansas City, Missouri, USA; b School of Medicine, University of Missouri–Kansas City, Kansas City, Missouri, USA

**Keywords:** autoimmune disease, heterotaxy-polysplenia syndrome, perinatal counseling, temporary pacing

## Abstract

Congenital complete heart block (CCHB) is a rare condition associated with significant fetal and neonatal mortality. It may present as an isolated conduction abnormality in association with autoimmune disease, or in conjunction with congenital heart defects including heterotaxy-polysplenia syndrome. Early prenatal diagnosis and coordinated perinatal care are critical for improving outcomes. We present 2 cases of prenatally diagnosed CCHB. The first occurred in a fetus with heterotaxy-polysplenia syndrome, and the second in the setting of maternal anti-SSA antibody exposure. Both fetuses developed progressive bradycardia, with heart rates declining to <50 beats/min, necessitating close fetal surveillance and individualized perinatal management. Preterm delivery and prompt placement of temporary pacing wires allowed initial stabilization before eventual definitive management, resulting in survival. The successful outcomes in these neonates highlight the critical role of multidisciplinary prenatal care, timely delivery planning, and early postnatal intervention in improving survival in fetuses diagnosed with CCHB.

## Graphical Abstract

**Figure:**
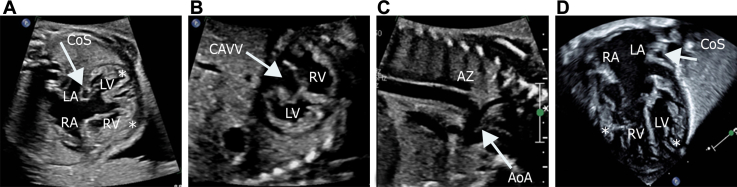

Congenital complete heart block (CCHB) is a rare and life-threatening fetal arrhythmia, with an incidence of 1 in 15,000 live births. It may occur in isolation or in association with structural heart disease or maternal autoimmune conditions. The natural history of CCHB varies widely, with fetal bradycardia often progressing during gestation, resulting in decreased cardiac output, hydrops fetalis, or even intrauterine demise in severe cases.^1^

**Take-Home Messages**

• Heterotaxy-polysplenia syndrome with CCHB presents additional surgical and prognostic complexity, often requiring escalation to transplantation.
• Fetal heart rate trends, Doppler changes, ventricular function, and fetal well-being should guide individualized timing of delivery.
• Immediate postnatal temporary pacing can stabilize critically bradycardic premature neonates and provide a bridge to definitive pacemaker placement.
• Effective prenatal counseling, anticipatory delivery planning and shared decision-making are essential to ensure that treatment goals align with the expectations and values of the family, maximizing the chances of achieving the best possible outcomes.

Although survival rates for isolated CCHB, often related to maternal anti-SSA/Ro antibodies, have increased after appropriate antenatal and perinatal management with steroids, intravenous immunoglobulin, sympathomimetics, and pacemaker implantation after delivery, cases associated with complex congenital heart disease, particularly heterotaxy-polysplenia syndrome, are challenging.^2^ Survival in CCHB depends heavily on early recognition, coordinated multidisciplinary planning, and prompt intervention. Despite advances in treatment options, significant morbidity and mortality exist.^3^

In this case series, we describe 2 fetuses diagnosed prenatally with CCHB: one associated with heterotaxy-polysplenia syndrome and complex cardiac anatomy, and the other occurring in the setting of maternal autoimmune disease with normal cardiac anatomy. Despite progressive fetal bradycardia, prematurity, and early signs of cardiovascular decompensation, both infants survived owing to comprehensive fetal surveillance, individualized delivery planning, and timely postnatal pacing support allowing for eventual definitive management. These cases exemplify how multidisciplinary teamwork and early postnatal stabilization strategies can result in successful outcomes, even in high-risk presentations of CCHB. Informed consent was obtained from the patients' families for the publication of this report.

## Case Presentation

### Patient 1

**Figure 1:**
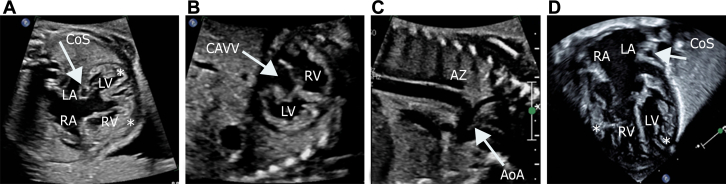
Fetal and Postnatal Transthoracic Echocardiogram Fetal echocardiogram demonstrating (A) 4-chamber view, (B) short-axis view of the ventricles, and (C) sagittal view of the aortic arch. (D) Postnatal 4-chamber echocardiographic view demonstrating the CAVV and ventricular noncompaction. Asterisks indicate noncompaction. AoA = aortic arch; AZ = azygos vein; CAVV = common atrioventricular valve; CoS = coronary sinus; LA = left atrium; LV = left ventricle; RA = right atrium; RV = right ventricle.

**Figure 2:**
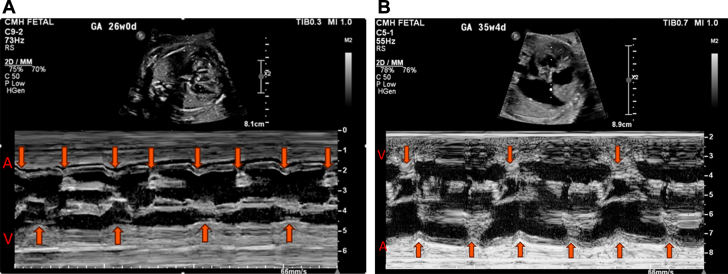
M-Mode Tracing Through the Atria and Ventricle Demonstrating Complete AV Block M-mode tracings showing complete heart block (A) at 26 weeks gestational age with ventricular rate of 60 beats/min and (B) progressive decline in heart rate at 35 weeks gestational age with ventricular rate of 50 beats/min.

A 26-year-old woman was referred to our fetal health center at 26 weeks gestational age (GA) for a fetal echocardiogram (FE). The FE demonstrated moderate cardiomegaly, congenital heart block–related bradycardia, a cardiac segmental anatomy of {S/A,D,S}, an unbalanced, right-dominant complete atrioventricular canal (Figure 1A and 1B), mildly crowded left ventricular outflow tract, mildly hypoplastic distal aortic arch and isthmus (Figure 1C), dysplastic pulmonary valve, and interrupted inferior vena cava with azygous continuation to a persistent left superior vena cava draining into a dilated coronary sinus (Figure 1A). In addition, the myocardium appeared thickened and hypertrabeculated, consistent with noncompaction (Figure 1D); biventricular systolic dysfunction was present with a small pericardial effusion. Ductus venosus Doppler was notable for a-wave reversal, however the umbilical artery and middle cerebral artery waveforms were normal. Maternal SSA and SSB antibodies were negative. Close follow-up with biweekly FEs demonstrated decreasing ventricular rates and systolic function, with worsening extracardiac Doppler (increased umbilical artery end-diastolic velocity). An urgent cesarian section was performed after follow-up FE at 35 weeks 3 days revealed decreasing fetal heart rate (FHR); the FHR was approximately 60 beats/min (Figure 2A) at 26 weeks GA but was 48 to 52 beats/min by 35 weeks GA (Figure 2B), with worsening ventricular function.

**Figure 3:**
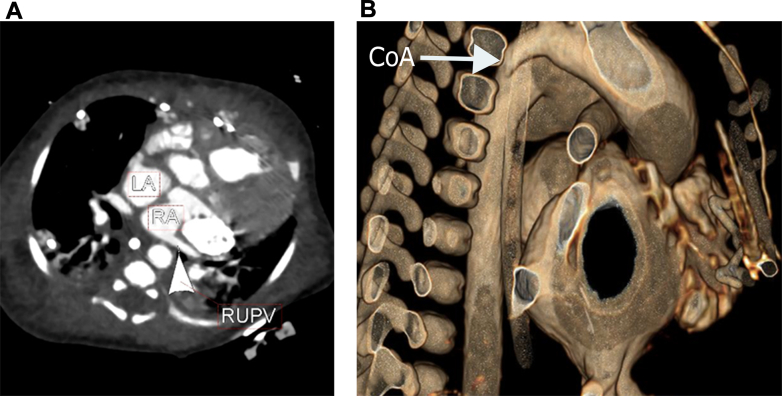
Computed Tomography Angiogram Demonstrating Venous and Arterial Anomalies (A) Computed tomography angiogram axial showing the right upper pulmonary vein (RUPV) connecting to the right atrium (RA) and (B) maximum-intensity projection reconstruction of the aortic arch showing the hypoplastic aortic isthmus (arrow). CoA = coarctation of aorta; LA = left atrium.

A female newborn was delivered, with a birth weight of 2,730 g and Apgar scores of 4, 4, and 8 at 1, 5, and 10 minutes, respectively. She was immediately transferred to the cardiovascular operating room for successful placement of temporary pacing wires. Postnatal echocardiogram and computed tomography angiogram confirmed the prenatal anatomic diagnosis and additionally showed partial anomalous pulmonary venous connection of the right upper pulmonary vein to the right atrium (Figure 3A) and coarctation of aorta (Figure 3B).

At 4 weeks of age, the patient underwent surgical repair including coarctation repair, patent ductus arteriosus ligation, and permanent pacemaker placement. Progressive biventricular dysfunction and bilateral outflow tract obstruction, as well as the presence of ventricular noncompaction and pacemaker dependency, resulted in listing for a heart transplantation at 2 months of age, with the patient eventually undergoing a successful transplant at 4 months of age. She is currently clinically stable at 3.5 years of age.

### Patient 2

**Figure 4:**
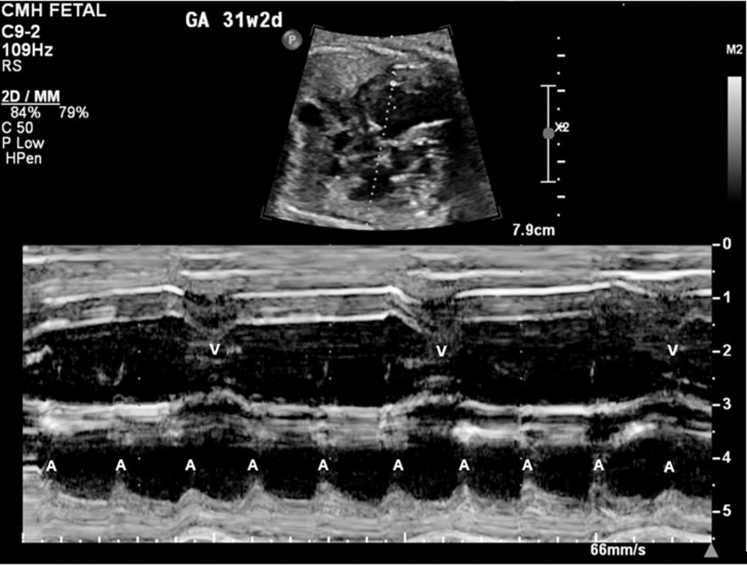
M-Mode Tracing Showing Complete Heart Block With a Ventricular Rate of 50 beats/min at 31-2/7 Weeks Gestational Age

**Figure 5:**
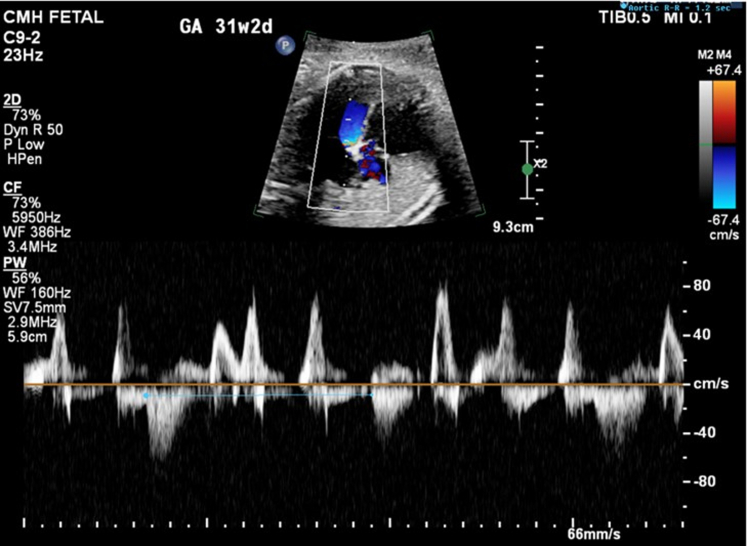
Doppler Interrogation of the Left Ventricular Mitral Valve Inflow and LVOT Flow The blue cursor is placed at the onset of aortic flow and measures an aortic R-R interval of 1.2 seconds, correlating to a fetal ventricular rate of 50 beats/min. The Doppler above the baseline is representative of the mitral valve inflow. There are both biphasic inflow Doppler patterns as well as high-velocity “a” waveforms seen corresponding to atrial contraction under conditions of abnormal ventricular filling properties. LVOT = left ventricular outflow tract.

**Figure 6:**
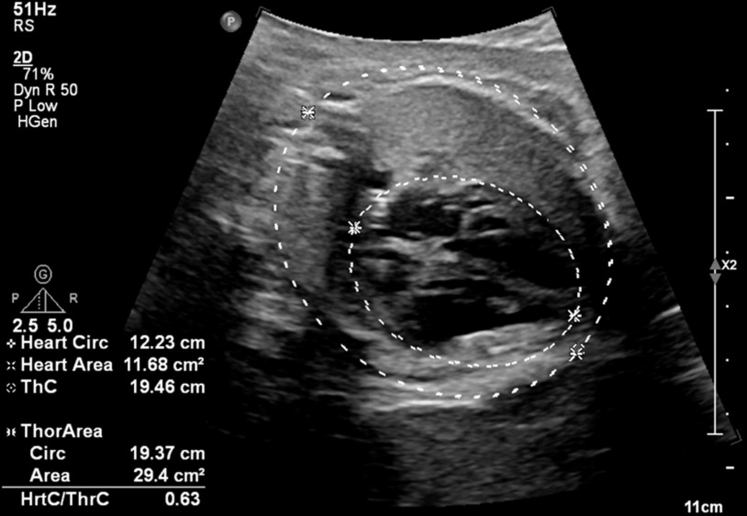
Cardiomegaly With a Cardiothoracic Ratio of 0.63 by Circumference and 0.40 by Area

A 23-year-old woman was referred to our fetal health center at 22-5/7 weeks gestation after a fetal anatomy scan at 21-5/7 weeks revealed CCHB with a ventricular rate of 55 beats/min. FHR was reported to be normal at 16 weeks GA. No family history of congenital or acquired heart disease, arrhythmias, sudden cardiac death, cardiomyopathy, or members with pacemaker or defibrillator was present. Maternal history was significant for a prior spontaneous first-trimester pregnancy loss, no major medical issues, and no medication intake other than prenatal vitamins. FE at 22-5/7 weeks revealed an FHR of 50 to 55 beats/min. Maternal work-up was positive for SSA antibodies. Dexamethasone and intravenous immunoglobulin were administered along with terbutaline, aiming at increasing the FHR. Weekly follow-up was established, and FE at 31-2/7 weeks GA showed declining FHR to 49 to 50 beats/min (Figures 4 and 5) with moderate cardiomegaly (Figure 6) and qualitatively normal biventricular function, with no evidence of hydrops fetalis.

**Figure 7:**
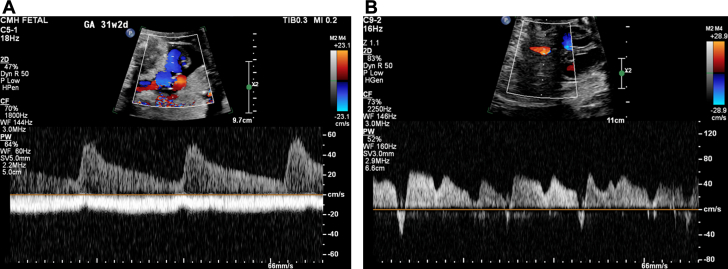
Abnormal Fetal Umbilical and Ductus Venous Doppler Waveforms (A) Umbilical venous (UV) Doppler below the baseline demonstrating UV pulsations. Normal umbilical arterial Doppler above the baseline aside from fetal bradycardia. (B) Abnormal ductus venosus Doppler pattern with a-wave reversal.

Owing to worsening fetal bradycardia and high clinical concern for intrauterine fetal demise based on abnormal umbilical venous pulsations (Figure 7A) and ductus venosus Doppler notable for a-wave reversal (Figure 7B), a cesarean section was performed at 31-3/7 weeks, resulting in the birth of a female with Apgar scores of 7 and 7 at 1 and 5 minutes, respectively, and a birth weight of 1,500 g. As initial ventricular escape rhythm at 35 to 45 beats/min showed no improvement with isoproterenol infusion, the newborn was promptly transferred to the cardiovascular operating room for successful placement of temporary pacing wires. After a 4-week stay in the cardiac intensive care unit, during which she was temporarily ventricular paced and demonstrated adequate somatic growth, she underwent permanent epicardial pacemaker placement at 1 month of age. The patient was discharged home 2 weeks later and remains clinically stable at 9 months of age, with the most recent echocardiogram demonstrating normal cardiac function.

## Discussion

CCHB linked to heterotaxy-polysplenia syndrome has been historically linked to poor survival outcomes, with early reports estimating survival rates as low as 10% to 25%, especially when associated with prematurity. However, recent advancements in prenatal diagnosis and neonatal management, together with early pacemaker placement, have improved the 1-year survival rate to approximately 60%.^2^ Heterotaxy-polysplenia syndrome can be associated with myocardial noncompaction, left ventricular outflow tract obstruction, and coarctation of the aorta, contributing to poorer prognosis despite pacemaker implantation.^3^ Our first case involved a combination of these infrequent structural abnormalities of heterotaxy-polysplenia syndrome in addition to congenital heart block, contributing to highly challenging management and a guarded prognosis.

Furthermore, heart transplantation in patients with heterotaxy syndrome presents unique surgical challenges given anatomical anomalies such as dextrocardia and systemic or pulmonary venous abnormalities. In addition, heterotaxy syndrome is an independent risk factor for poorer post-transplant outcomes, including increased postoperative mortality and lower survival rates compared with other congenital heart disease patients undergoing heart transplantation.^4^

In contrast, autoimmune-mediated CCHB typically occurs in the setting of normal cardiac anatomy and results from transplacental passage of maternal SSA (Ro) and/or SSB (La) antibodies. These antibodies induce an inflammatory response in the fetal myocardium, leading to fibrosis and scarring of the conduction system, which results in heart block. Importantly, higher maternal anti-SSA antibody titers and, more recently, the presence of anti-Ro52 antibodies have been associated with an increased risk of fetal CCHB development and progression.^5^ Although the prognosis for isolated autoimmune-mediated CCHB is better than for cases associated with complex congenital heart disease, the condition still carries significant risks both prenatally and postnatally. The use of glucocorticoids such as dexamethasone and intravenous immunoglobulin in autoimmune-mediated CCHB remains controversial. Although these therapies are frequently administered in clinical practice, evidence regarding their impact on fetal outcomes is limited and inconsistent. Corticosteroids may not reverse established complete atrioventricular block, although intravenous immunoglobulin has been suggested for use in cases associated with myocardial dysfunction, immature lungs, or signs of fibroelastosis. Shared decision-making and individualized risk-benefit evaluation are essential in these cases.^6^ In cases of FHR <55 beats/min, beta sympathomimetics such as terbutaline may be administered to increase ventricular rates, particularly in isolated CCHB cases where it appears to have a more pronounced effect.^7^ Emerging therapies for high-risk cases of CCHB, including fetuses with hydrops and ventricular rates <45 beats/min, have explored the feasibility of in utero pacemaker implantation. Although still experimental and limited to case reports, transplacental pacing in select fetuses has been reported as a potential rescue option. Future advances in fetal pacing technology may provide additional tools in the management of life-threatening CCHB.^1^

Prematurity introduces additional risks in CCHB, with affected neonates more frequently requiring temporary pacing prior to permanent pacemaker implantation. Worse perinatal outcomes have been associated with prematurity, lower FHR (<50 beats/min), rapid progression of bradycardia, declining ventricular function, atrioventricular valve regurgitation, and the presence of hydrops.^8^

Given the limited therapeutic options available for managing CCHB in utero and the guarded prognosis, it is essential to adopt a shared decision-making approach when discussing prenatal and perinatal interventions. Close follow-up is recommended, and treatment decisions should be continually reassessed throughout pregnancy to align with the evolving clinical data and maternal expectations. Current expert consensus guidelines recommend weekly or biweekly FEs to monitor atrioventricular intervals, ventricular rates, and signs of fetal compromise.^7^ A cardiovascular profile score is an important adjunct to monitor fetal cardiac function, integrating Doppler and functional assessments to guide management decisions.^9^ Delivery planning with appropriate neonatal resuscitation at a dedicated cardiac center are crucial components of CCHB management, as these infants are at high risk for hemodynamic instability from poor cardiac output and bradycardia. Immediate interventions such as ascertaining the need for adequate oxygenation and ventilation, inotropic support, sedation, and neuromuscular blockade may be necessary to minimize oxygen consumption and stabilize the neonate for transport to the cardiovascular operating room.^10^ Emergency temporary pacing is often required, followed by permanent pacemaker implantation.^8^ In our 2 clinical cases, meticulous planning and multidisciplinary teamwork between fetal cardiology, maternal fetal medicine, neonatology, cardiac electrophysiology, cardiovascular surgery, anesthesiology, and supporting staff resulted in early stabilization of the neonates, followed by immediate and successful temporary pacemaker lead placement. This served as a bridge to permanent pacemaker implantation and eventual definitive management, resulting in survival of both our patients.

## Conclusions

CCHB is a rare but serious condition that can be detected in utero, with limited definitive fetal treatment options. Prognosis remains guarded, with significant morbidity and mortality risks throughout both fetal and neonatal life. Mortality rates are even higher in CCHB occurring in the setting of heterotaxy-polysplenia syndrome, associated with outflow tract obstruction and ventricular noncompaction. Survival rates worsen with additional risk factors such as prematurity, FHR <55 beats/min, and ventricular dysfunction. Regardless of etiology, advances in prenatal diagnosis, close fetal surveillance, structured delivery planning, and prompt postnatal interventions, including pacing and listing for heart transplantation in select cases, may improve survival.

## Funding Support and Author Disclosures

The authors have reported that they have no relationships relevant to the contents of this paper to disclose.
